# A Modular BAM Complex in the Outer Membrane of the α-Proteobacterium *Caulobacter crescentus*

**DOI:** 10.1371/journal.pone.0008619

**Published:** 2010-01-08

**Authors:** Khatira Anwari, Sebastian Poggio, Andrew Perry, Xenia Gatsos, Sri Harsha Ramarathinam, Nicholas A. Williamson, Nicholas Noinaj, Susan Buchanan, Kipros Gabriel, Anthony W. Purcell, Christine Jacobs-Wagner, Trevor Lithgow

**Affiliations:** 1 Department of Biochemistry and Molecular Biology, Monash University, Melbourne, Australia; 2 Department of Molecular, Cellular and Developmental Biology and Microbial Pathogenesis Section, Yale University, New Haven, Connecticut, United States of America; 3 Department of Biochemistry and Molecular Biology, University of Melbourne, Parkville, Australia; 4 National Institute of Diabetes and Digestive and Kidney Diseases, National Institutes of Health, Bethesda, Maryland, United States of America; 5 Howard Hughes Medical Institute, Yale University, New Haven, Connecticut, United States of America; CNRS UMR6543, Université de Nice, Sophia Antipolis, France

## Abstract

Mitochondria are organelles derived from an intracellular α-proteobacterium. The biogenesis of mitochondria relies on the assembly of β-barrel proteins into the mitochondrial outer membrane, a process inherited from the bacterial ancestor. *Caulobacter crescentus* is an α-proteobacterium, and the BAM (β-barrel assembly machinery) complex was purified and characterized from this model organism. Like the mitochondrial sorting and assembly machinery complex, we find the BAM complex to be modular in nature. A ∼150 kDa core BAM complex containing BamA, BamB, BamD, and BamE associates with additional modules in the outer membrane. One of these modules, Pal, is a lipoprotein that provides a means for anchorage to the peptidoglycan layer of the cell wall. We suggest the modular design of the BAM complex facilitates access to substrates from the protein translocase in the inner membrane.

## Introduction

Mitochondria are essential organelles, derived in the first eukaryotes from intracellular bacterial symbionts. Phylogenetic reconstructions show that these endosymbionts were of α-proteobacterial descent, and key aspects of mitochondrial biology evolved from the bacterial ancestor [1]–[4]. The outer membranes of mitochondria have β-barrel proteins and these are assembled by the SAM complex. An essential feature of the SAM complex is its modular nature. Protein subunits that are important, but not essential, for the core function of β-barrel assembly can be titrated from the complex by increasing the concentration of detergent used in purification. In this way the essential protein “modules” such as Mdm10 and Mim1, can be selectively removed from the holo-SAM complex [5]–[10]. This modular machine in the outer membrane receives its substrates via small TIM chaperones that link the protein translocation device (the TOM complex) with the SAM complex [11], [12].

A central and essential component of the mitochondrial SAM complex, Sam50, is a member of the Omp85 family of proteins [13]–[16]. All bacteria with outer membranes have an Omp85 protein [15], [17], with studies in both *Neisseria meningitidis* and *Escherichia coli* showing that the Omp85 protein mediates the assembly of β-barrel proteins into the outer membrane [18]–[20]. Thus, mitochondria have retained from their α-proteobacterial ancestry the machinery to assemble proteins into the organelle's outer membrane. However, there are significant differences in the subunit composition of the mitochondrial SAM complex and the BAM complex in the model γ-proteobacterium *E. coli*.

Recent studies in *E. coli* have shown that the Omp85 protein, now referred to as BamA, is the central component of a complex that also contains four lipoprotein partners: BamB, BamC, BamD and BamE [20]–[23]. BamB (YfgL) interacts with BamA and the interaction site has been mapped by mutagenesis [24]; BamB is predicted to have a β-propeller structure and the interaction might be mediated through unpaired β-strands in BamA and BamB [24], [25]. In *Serratia marcescens*, BamC (NlpB) has been implicated in the regulation of cell motility [26], but little is known of its function. The gene encoding BamD (*yfiO*) is essential for cell viability in *E. coli* and BamD and BamC interact directly [21]. BamD is predicted to be rich in tetratricopeptide repeat (TPR) structure [25], a feature it shares with the mitochondrial receptor for β-barrel proteins [23], [27]. BamE (SmpA/OmlA) is needed for outer membrane integrity in *E. coli*, *Pseudomonas aeruginosa* and *Salmonella enterica* [22], [28], [29]. Despite a high degree of sequence conservation across the major groups of proteobacteria, none of these lipoproteins have homologs in eukaryotes and thus must be absent from the machinery that assembles β-barrel proteins in mitochondria [25].

We sought to determine if the BAM complex, like the mitochondrial SAM complex, might be modular in structure, with novel modules conferring important properties to the holo-BAM complex. Because of the ancestral link between mitochondria and α-proteobacteria, we developed methods to characterize the BAM complex from *Caulobacter crescentus*, using native-gel electrophoresis and immunoprecipitation with an antiserum directed against the BamA subunit. After immunoprecipitation, mass spectrometry revealed the presence of BamB, BamD and Bam E subunits. In addition, the α-proteobacterial BAM complex has a novel subunit, Pal, with a characteristic OmpA-like domain that binds to the peptidoglycan layer rendering Pal immobile in the outer membrane *in vivo*. We suggest that this anchorage of Pal minimizes the separation between the outer and inner membrane, thereby assisting the BAM complex in accessing substrate proteins transported through the cytoplasmic membrane.

## Methods

### Strains and Growth

The wild-type strain used in these studies is CB15N. The construction of the Pal-mCherry-producing strain (CJW2965) is described [30]. The Pal depletion strain (CJW3131) was obtained by single crossover of the suicide vector pXCC3229. This integrates a xylose-inducible promoter upstream of the *pal* open-reading frame (CC3229). The suicide vector pXCC3229 was obtained by cloning an internal fragment of CC3229 into pXMCS2. This internal fragment was obtained by PCR using CC3229F2 (5′-CACATATGAGC TTCGACACCCAGCGC-3′) and CC3229R2 (5′-CAGGATCCAGGAAGTCGCGCACGGCGTTG-3′) primers. For cell growth, peptone yeast extract (PYE) was prepared as previously described [31]. All cultures were grown under aerobic conditions at 30°C shaking at 120 rpm in 5 L baffled flasks to an optical density (OD)_600_ of 0.7 units/mL.

### Subcellular Fractionation

A method was developed to purify outer membrane vesicles from *C. crescentus*. Cell pellets from 1L PYE cultures were resuspended in 50 mL (10 mM Tris, 0.75 M sucrose, pH 7.5) followed by the addition of 100 µg/mL lysozyme and protease inhibitor cocktail (Roche), and 1.5 mM EDTA (pH 7.5) was then added drop-wise to a final concentration of 0.5 mM EDTA. Cells were disrupted by two passages through a French pressure cell at 15,000 psi and cellular debris removed by centrifugation. Membranes were collected (42,000 rpm, 1 h, Beckman Ti45 rotor) and resuspended in 25% (w/v) sucrose, 5 mM EDTA (pH 7.5) and fractionated on a six-step sucrose gradient (35∶40∶45∶50∶55∶60% (w/v) sucrose in 5 mM EDTA, pH 7.5) by centrifugation (Beckman SW41 rotor, 34000 rpm, 40 h). Outer membrane vesicles were collected with an ISCO density gradient fractionator. All steps were performed at 4°C and outer membranes were stored at −80°C.

### Immunological Methods and Electrophoresis

To generate the antibody recognizing BamA, BL21(DE3) cells were transformed to express a hexahistidine-tagged BamA and the protein purified from inclusion bodies. Protein purity was checked by SDS-PAGE and the protein preparation was injected into rabbits. The purity of the antigen and specificity of the serum is documented in Figure S1. Anti-mCherry was purchased from Clontech Laboratories.

For blue native polyacrylamide gel electrophoresis (BN-PAGE), purified outer membrane vesicles were washed (10 mM Tris, 1 mM EDTA, pH 7.5) and recovered by ultracentrifugation. Outer membranes were resuspended in ACA750 buffer (750 mM *n*-aminocaproic acid, 50 mM Bis-Tris, 0.5 mM Na_2_EDTA, pH 7.0) as previously described [32] and solubilised with dodecyl-maltoside (*N*-dodecyl-β-maltoside, Anatrace) by vortexing every 5 min for 20 min. Samples were pre-cleared (310, 000×g at 4°C for 20 min). Samples were analysed by BN-PAGE on 5–16% (w/v) polyacrylamide gels [33] using gel buffers as previously described [32].

For immunoprecipitations, purified outer membranes (approximately 800 µg protein) were resuspended in ACA750 buffer and dodecyl-maltoside as for BN-PAGE, and centrifuged (25,000×g, 4°C for 10 min). Cross-linked IgG-Protein A Sepharose beads (Sigma) were added to solubilised outer membranes and incubated for 3 h at 4°C with shaking. After six washes with PBS containing 0.1% (w/v) dodecyl-maltoside (final concentration), a final wash with PBS was used before eluting the immunoprecipitates with 2× SDS- Laemmli sample buffer. As a negative control, immunoprecipitations were also performed using pre-immune serum collected from the rabbits later immunised with BamA.

### Microscopy

Microscopy images were obtained using NIKON E80i equipped with a Hamamatsu ORCA ERII or Andor iXon^EM^+ CCD camera. Cells were grown to an OD_660_ between 0.2 and 0.3 units/mL, washed in minimal (M2G) medium and immobilized on an agarose-padded slide. When required, chloramphenicol was added to the culture 30 min before observation and to the agarose pad at a final concentration of 20 µg/ml. Images were taken and processed by Metamorph 7.1.4 software. For photobleaching experiments, a Photonic Instrument Micropoint Laser system was used with a 552-nm Laser Dye. Kymographs were constructed using Metamorph 7.1.4 software.

### Mass Spectrometry

Protein-containing bands from SDS gels were excised manually and destained using 100 mM TEAB (Triethylammonium bicarbonate, pH 8.5) in 50% (v/v) acetonitrile. Gel pieces were dehydrated using 100% (v/v) acetonitrile and air-dried before adding 20 mM dithiothreitol at 60°C for 1 h and then 100 mM iodoacetamide at room temperature for 30 min in the dark. Gel pieces were washed with destaining solution and dehydrated as above followed by rehydration in the presence of 250 ng sequencing grade trypsin (Sigma) in 50 mM TEAB. Digests were allowed to proceed overnight at 37°C. Tryptic peptides were extracted using 0.1% (v/v) formic acid and analysed by liquid chromatography-tandem MS (LC-MS/MS) using a Shimadzu Prominence nano flow HPLC and an Applied Biosystems Q-STAR ELITE mass spectrometer as previously described [34].

### Homology Model

A structure-based sequence alignment was performed in STRAP [35] using sequences and coordinates for YiaD (PDB code 2K1S), RmpM (PDB code 1R1M), and Pal (PDB code 2AIZ) and aligning these to the Q9A3H5 sequence. The results were visualized in JalView [36] and the figure was prepared using Adobe Illustrator.

## Results

### The BamA Protein in α-Proteobacteria

**Figure 1:**
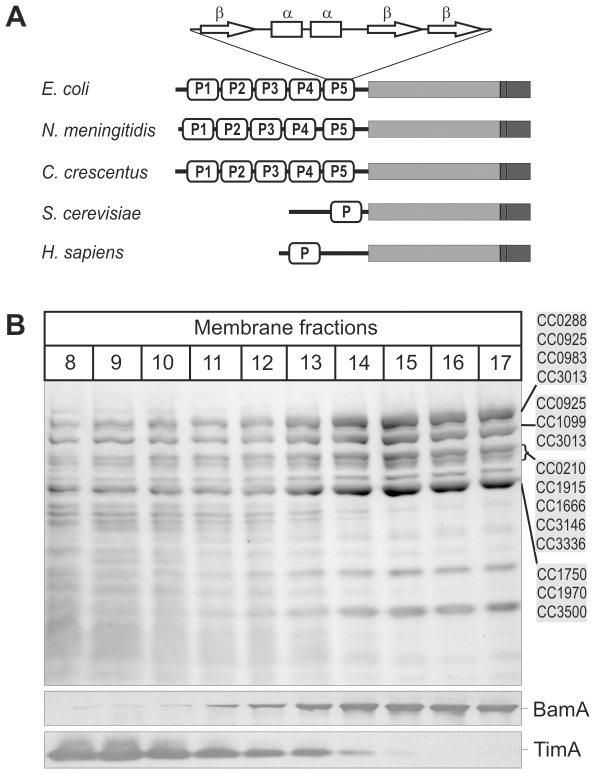
BamA in *Caulobacter crescentus*. (A) The domain structure of BamA. The 5 predicted POTRA domains (P1–P5) are shown in white, with the secondary structure β-α-α-β-β indicated [56]–[58] and the predicted β-barrel domain in grey. The defining “Omp85” motif [63] is shaded a darker grey. The mitochondrial Sam50 proteins from yeast (*S. cerevisiae*) and humans (*H. sapiens*) are shown for comparison. (B) Membranes from wild-type *C. crescentus* were fractionated on sucrose gradient and analysed by SDS-PAGE. Coomassie Brilliant Blue staining (upper panel) reveals separation of the membrane protein profiles and immunoblotting (lower panel) for the inner membrane protein TimA and the outer membrane protein BamA. Mass spectrometry of the major protein bands indicated revealed identities of fifteen proteins (CC0288, CC0925, etc) annotated as TonB-dependent receptors [37].

In keeping with a common ancestry, the mitochondrial Sam50 and α-proteobacterial BamA cluster together in phylogenetic analyses [15]. Whereas the mitochondrial Sam50 has a truncated N-terminal domain (Figure 1A), the BamA protein from *C. crescentus* and other α-proteobacteria conform to the basic domain structure seen in all other BamA proteins: a signal sequence for secretion via the SecYEG machinery, five predicted POTRA domains and a C-terminal β-barrel that would be embedded in the outer membrane (Figure 1A). Thus, truncation of the POTRA domains is a mitochondria-specific and not a lineage-specific adaptation.

We developed a method to prepare and purify outer membrane vesicles from *C. crescentus*. Sucrose gradients separate the bacterial membranes into two populations, and the major membrane proteins in fractions 15–17 are all TonB-dependent receptors (Figure 1B). The identities of fifteen of these proteins were confirmed by mass spectrometry and are indicated in Figure 1B. In *C. crescentus*, TonB-dependent receptors appear to be the dominant means of substrate transport across the outer membrane and the *C. crescentus* genome codes for an extraordinary sixty-seven TonB-dependent receptors, leading to the suggestion that a repertoire of diverse active transport pathways can be established in the outer membrane of this bacterium [37], [38]. Immunoblotting with an antibody specific for the *C. crescentus* BamA showed the protein present in these outer membrane fractions (Figure 1B, lower panel).

### The BAM Complex Is a Modular Machine

**Figure 2:**
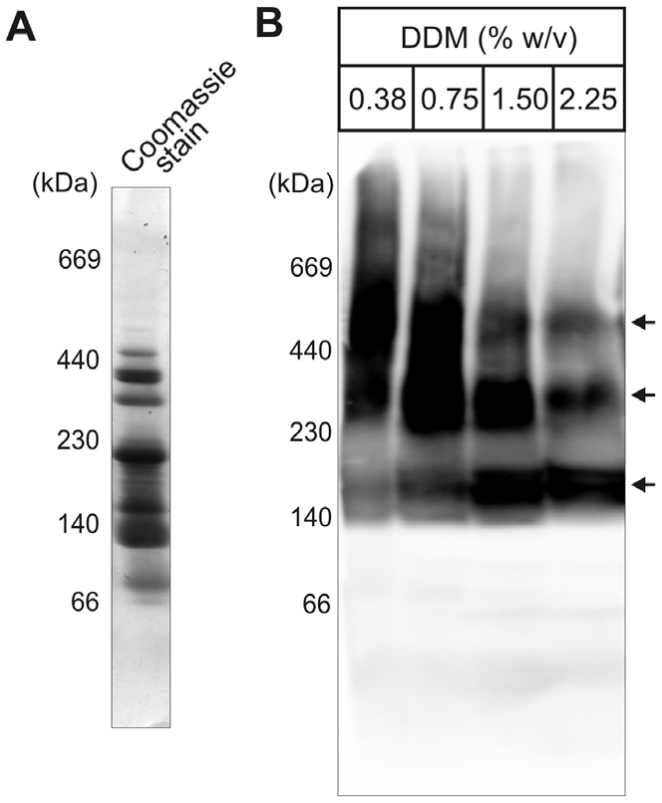
The BAM complex is modular. (A) Outer membrane vesicles (100 µg protein) were solubilised with 0.38% (w/v) dodecyl-maltoside and loaded for analysis by blue native-PAGE. The Coomassie Brilliant Blue-stained gel shows the major outer membrane protein complexes. (B) Outer membrane vesicles (100 µg protein per lane) solubilised with the indicated concentrations of dodecyl-maltoside, separated by blue native-PAGE and analysed by immuno-staining with an antiserum to BamA. The migration positions of the molecular weight markers are shown. Arrows indicate three modular forms of the BAM complex.

To determine whether or not the BAM complex in *C. crescentus* is modular, outer membrane vesicles were titrated with the non-ionic detergent dodecyl-maltoside and analysed by blue native polyacrylamide gel electrophoresis (BN-PAGE). A Coomassie-stained gel shows the major outer membrane proteins migrate in a clear pattern as well focussed complexes by this procedure (Figure 2A). Antibodies to BamA reveal the BAM complex is ∼500 kDa in size, but that it dissociates to a complex of ∼300 kDa and to a smaller core complex of ∼150 kDa with increasing concentration of detergent (Figure 2B).

**Figure 3:**
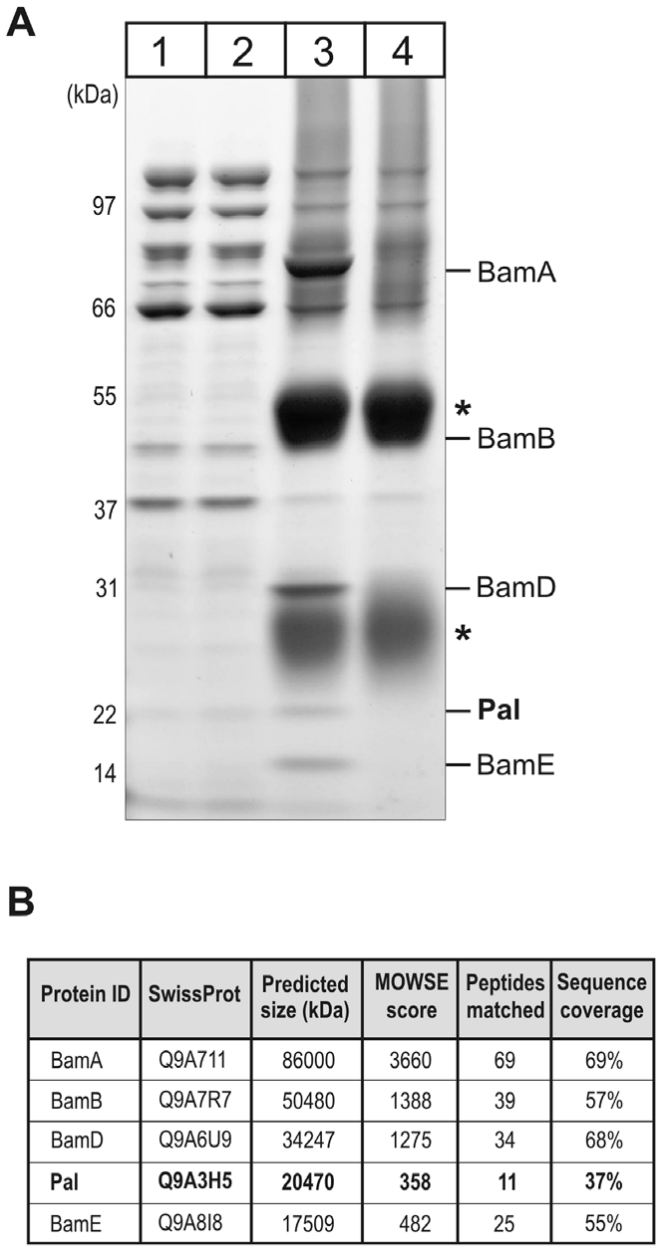
Subunit composition of the BAM complex in *C. crescentus*. (A) Outer membrane vesicles (800 µg protein) were solubilised with 0.75% (w/v) dodecyl-maltoside and the BAM complex immunoprecipitated with an antiserum recognizing the BamA subunit (lane 3) or preimmune serum (lane 4). Samples corresponding to 8 µg protein of total outer membranes (Lane 1), 8 µg protein of unbound fraction (Lane 2) and all of the material immunoprecipitated (Lane 3, 4) are shown. Asterisks indicate the IgG heavy and light chains, with the migration positions of the molecular markers shown in kDa. (B) Mass spectrometry data summarizing the identification of immunoprecipitated proteins (see Methods). In addition to the number of high confidence peptides identified by MS/MS data and sequence coverage; a MOWSE score is included, which is a probabilistic score that indicates the match of the experimental peptide precursor masses (peptide mass fingerprint) to the sequence of a candidate parental protein. Typically a MOWSE score >75 is considered as significant [64].

In *E. coli*, BamA is associated with four lipoprotein partners, and analysis of the *C. crescentus* genome revealed genes that could encode three of these lipoproteins: BamB (CC1653), BamD (CC1984) and BamE (CC1365) [25]. In order to determine the subunit composition of the BAM complex in *C. crescentus*, outer membrane vesicles were solubilised in 0.75% (w/v) dodecyl-maltoside and the solubilised membrane proteins incubated with the BamA antiserum. Immunoprecipitation shows four proteins specifically precipitated together with BamA (Figure 3A). The bioinformatic predictions were validated by the identification of the lipoproteins BamB, BamD and BamE by mass spectrometry (Figure 3B). In addition, a novel lipoprotein was identified, annotated in the genome of *C. crescentus* as CC3229 – “OmpA family protein”. For reasons detailed below we refer to the protein as Pal (Peptidoglycan-associated lipoprotein). Pal is a protein of ∼20 kDa with closely-related sequences in various bacteria showing the characteristic sequence features of a lipoprotein [39] and a C-terminal OmpA-like domain (Pfam: PF00691), found in peptidoglycan binding proteins [40].

**Figure 4:**
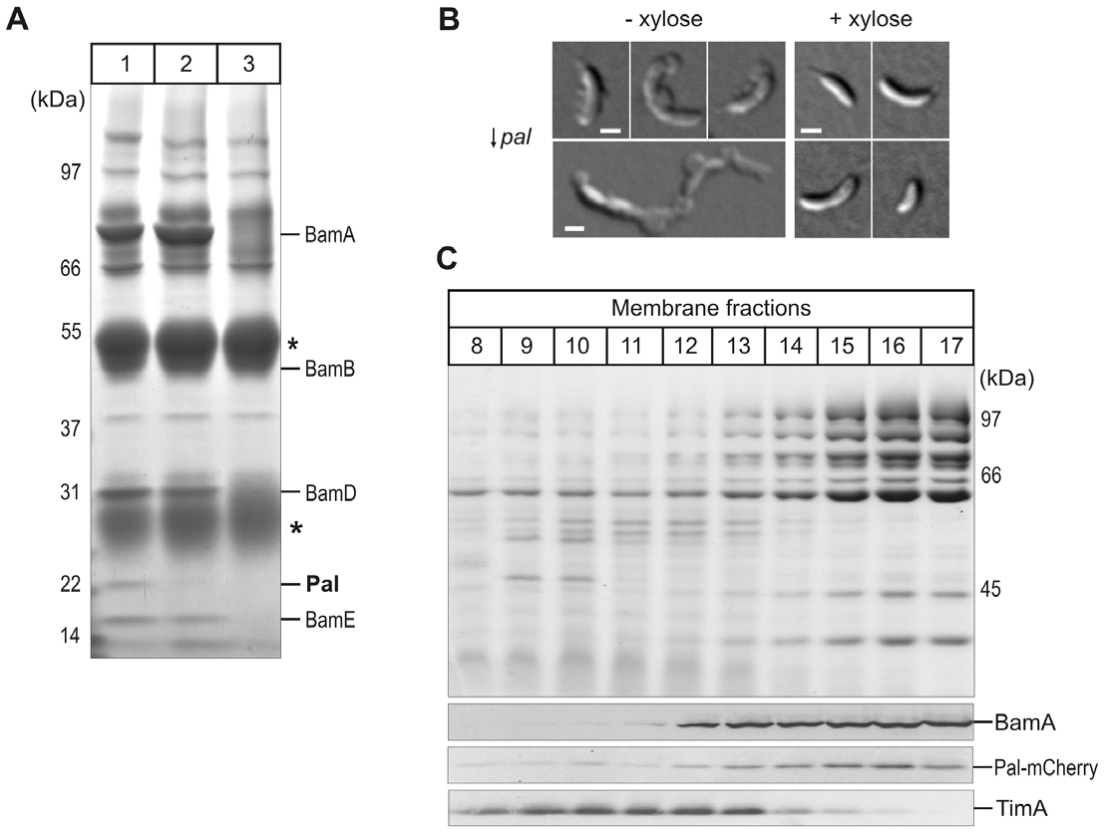
Pal is an essential outer membrane protein, associated with the BAM complex. (A) BAM complex was immunoprecipitated using BamA antiserum added to outer membrane vesicles that were solubilised with 0.75% (w/v) (Lane 1) and 2.25% (w/v) (Lane 2) dodecyl-maltoside. Immunoprecipitate obtained with preimmune serum was loaded in Lane 3. Asterisks indicate the IgG heavy and light chains, with the migration positions of the molecular markers shown in kDa. (B) Cells with the *pal* gene under the control of a xylose-inducible promoter (↓ *pal*) were grown in the presence (right montage) and absence (left montage) of xylose (0.3% [w/v]) for 10 hrs. Outer membrane blebs that form predominantly from the division site or cell poles are evident only in the Pal-depleted cells. Scale bars (white) represent 1 micrometer. (C) Membranes were fractionated on sucrose gradient and analysed by SDS-PAGE. Coomassie Brilliant Blue staining (upper panel) reveals separation of the membrane protein profiles and immunoblotting (lower panel) for the inner membrane protein TimA and the outer membrane protein BamA, and the mCherry epitope to determine the location of Pal.

To identify the subunits released from the BAM complex with increasing detergent stringency, outer membranes were solubilised either in low [0.75% (w/v)] or high [2.25% (w/v)] concentration of dodecyl-maltoside and incubated with the BamA antiserum for immunoprecipitation (Figure 4A). Pal is less-tightly attached to the core BAM complex than other subunits as it dissociates from the BAM complex at higher detergent concentrations (Fig. 4A, lane 2).

Attempts to delete the gene encoding Pal suggest that it is an essential gene in *C. crescentus* (data not shown). We therefore constructed a strain in which the *pal* gene was under the control of a xylose-inducible promoter (see Methods): the resultant ↓*pal* strain required xylose for growth, consistent with *pal* being an essential gene. Microscopic examination of a freshly obtained Pal-depletion strain growing in the absence of xylose (↓*pal*) often displayed vesicles and blebs that mainly formed from the poles or division site (Figure 4B). This is consistent with a previous report on the phenotype of Pal-depleted *E. coli* [41] .

As a subunit of the BAM complex, Pal would be expected in the outer membrane. To test for its subcellular location, we used a strain expressing a Pal-mCherry fusion [30]. The viability of the strain demonstrates that the Pal-mCherry fusion is functional. Sucrose density gradient fractionation of membrane vesicles revealed that Pal is associated with outer membranes (Figure 4C), and epifluorescence microscopy of Pal-mCherry [30] shows a halo of fluorescence consistent with the mCherry fluorescent tag being exposed to the periplasm [30], [42].

**Figure 5:**
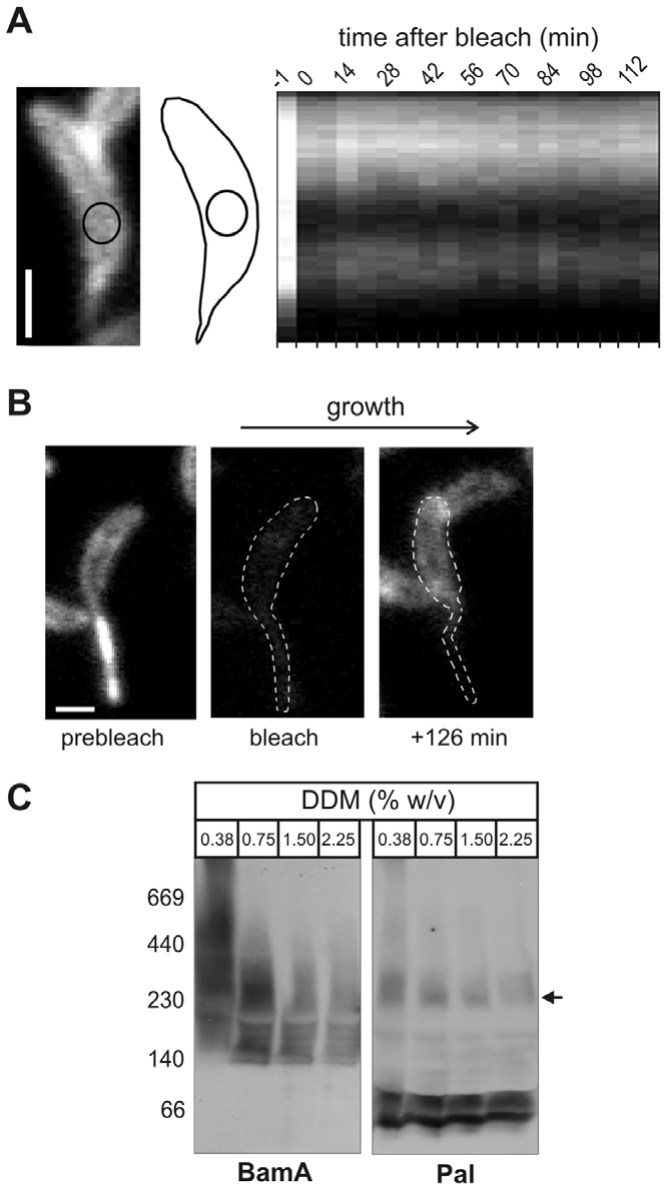
Pal anchors the BAM complex to the peptidoglycan layer of the cell wall. (A) Photobleaching analysis of Pal-mCherry dynamics required pre-treatment with chloramphenicol to block protein synthesis, and cells expressing Pal-mCherry were immobilized on an agarose-padded slide containing chloramphenicol. The left panel shows a representative cell in which the circle indicates the region that will be photobleached. The right panel shows a kymograph representation of Pal-mCherry fluorescence intensity along the cell axis before (−1 min) and following photobleaching. (B) Fluorescence micrographs showing a cell expressing Pal-mCherry before photobleaching (left), right after being completely photobleached (middle) and after 126 min of growth (right). (C) Outer membrane vesicles (100 µg protein per lane) from cells expressing Pal-mCherry were solubilised with dodecyl-maltoside and analysed by blue native-PAGE and immuno-staining with antisera recognizing either Pal or BamA. The migration position of molecular markers (kDa) are shown, arrow indicates the 300 kDa form of the BAM complex. Scale bars (white) represent 1 micrometer.

Purified Pal from *E. coli* (*Ec*Pal) binds peptidoglycan-related oligopeptides *in vitro* [39], demonstrating the possibility that Pal could dock the outer membrane to the peptidoglycan layer *in vivo*. To test this, we measured the dynamics of the Pal-mCherry fusion *in vivo* with time-lapse sequences of Pal-mCherry fluorescence, both before and after photobleaching a small region within cells treated with chloramphenicol to block protein synthesis. Kymographs were constructed from the series of micrographs to display the integrated fluorescence intensity along the long cell axis as a function of time [42]. These kymographs showed that no fluorescence recovery was observed after 2 hrs in all cells photobleached (n = 17; Figure 5A), indicating that Pal-mCherry does not move laterally in the outer membrane. By contrast, we have shown that under identical conditions, the recovery time of the integral membrane-bound Penicillin-Binding protein 3 (PBP3) is in the order of tens of seconds [42], and for soluble proteins such as cytoplasmic GFP, the recovery is immediate (data not shown). As further evidence to the relatively static location of Pal, cells expressing Pal-mCherry were completely bleached, and then allowed to grow and synthesize new Pal-mCherry. Time-lapse imaging showed that newly synthesized Pal-mCherry appeared around the cell body during growth (Figure 5B, right panel). In contrast, no Pal-mCherry signal appeared in the stalk. This suggests that newly-synthesized Pal is added predominantly where new peptidoglycan is incorporated; the stalk is made of old peptidoglycan, with stalk elongation proceeding from the cell body [43], [44].

BN-PAGE analysis shows that Pal is found in two outer membrane complexes (i) an ∼300 kDa complex co-migrating with BamA and (ii) a set of 70–120 kDa complexes which do not correspond to complexes containing BamA. Immunoblots using anti-BamA and anti-mCherry sera show that Pal is not associated with the ∼150 kDa forms of the BAM complex. Consistent with immunoprecipitation data (Figure 4A), at higher detergent concentrations BN-PAGE shows Pal is dissociated from the core BAM complex (Figure 5C).

**Figure 6:**
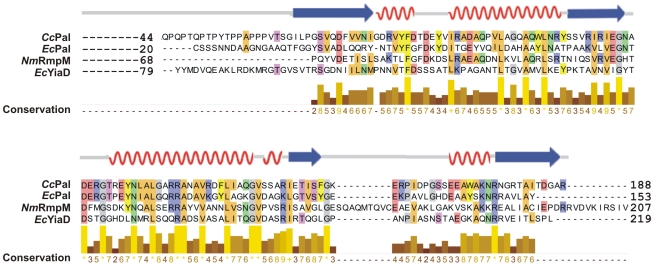
Structural homology model for CC3229 as the Pal from *C. crescentus*. The homology model is based on the known structures of Pal (PDB code 2AIZ) and YiaD (PDB code 2K1S) from *E. coli*, and RmpM (PDB code 1R1M) from *N. meningitidis*. Note that YiaD, *Cc*Pal and RmpM have N-terminal extensions that are not resolved in the crystal structures. Residues that show at least 50% conservation in structure are colour coded according to their properties. Below the sequence alignment is the conservation plot, which is based on a 1–10 scale with completely conserved residues highlighted with yellow bars and asterisks.

Structural modeling of Pal suggests it is closely-related to three lipoproteins for which crystal structures have been solved: YiaD (PDB code 2K1S) and *Ec*Pal [39] from *E. coli* and RmpM from *N. meningitidis* [40]. The *C. crescentus* Pal is sufficiently similar to these proteins that a structural model could be built with confidence (Figure 6, “*Cc*Pal”).

## Discussion

Pal is a lipoprotein, with a previous analysis by Edman sequencing showing the protein to be processed at a conserved cysteine that is modified with a lipid group [45]. This lipoprotein has biomedical importance, with Pal acting as an agonist for a specific isoform of Toll-like receptor (TLR2), thereby activating inflammation through cytokine release from macrophages [46]. Decreased expression levels of the Pal protein has been reported to impact on bacterial virulence [47], with similar findings reported for loss-of-function mutants lacking *yfgL* and *smpA* [29], [48], [49]: these genes encode the BamB and BamE lipoprotein subunits of the BAM complex. Decreased expression of Pal leads to outer membrane morphology defects providing a molecular explanation to the decreased virulence of Pal-depleted bacteria.

Pal has an OmpA-type peptidoglycan binding domain [40]. These domains are found in many outer and inner membrane proteins and serve to bind together structures within the bacterial cell wall. For example, the OmpA-type domain of MotB is essential to anchor the flagellum within the wall to ensure that flagellar motion translates into motility [50], [51]. Chimeric proteins have been used to show that the Pal OmpA-type domain can replace the equivalent domain in MotA to enable function of the flagellum [52]. Thus, Pal serves as an anchor, and anchorage will be bestowed to whichever protein complex Pal selectively binds. After solubilisation of outer membranes with detergent, immunoprecipitation analysis proved Pal and three other lipoproteins (BamB, BamD and BamE) are bound to BamA. When the same solubilisation conditions are used and analyzed by blue-native PAGE, these four lipoproteins: Pal, BamB, BamD and BamE are associated with BamA in the 300 kDa BAM holo-complex. More stringent conditions cause Pal to be released from the core BAM complex. Thus, Pal should be considered a module of the BAM complex.

Cryo-electron microscopy of *E. coli* and *Pseudomonas aeruginosa* suggest that the distance between the outer and inner membranes can vary considerably and is closest in regions where no periplasmic “space” exists, where the two membranes are closely apposed to the peptidoglycan layer [53]–[55]. The model proposed from these studies suggests that proteins anchoring membrane to peptidoglycan stabilize regions of relatively close proximity between the outer and inner membranes. While speculative at this stage, measurements of the POTRA domains in BamA [56]–[58] have led to the suggestion that the POTRA domain might span the distance to the cytoplasmic (inner) membrane, to assist in capturing substrates during translocation into the periplasm [58]. The Pal module of the BAM complex could assist in minimizing the distance the POTRA domains are required to span from the outer membrane to the sites of protein substrate entry into the periplasm.

While this manuscript was in preparation, Volokhina and co-workers reported that RmpM associates with the BAM complex in *Neisseria meningitidis* [59]. A study from Sauri et al investigating the transport pathway of the beta-barrel protein Hbp in *E. coli* found a translocation intermediate cross-linked to BamA and BamB, the periplasmic chaperone SurA and OmpA [60]. Further work is needed to fully understand the subunit composition of BAM holo-complexes, but the evidence so far suggests a generalized role for OmpA-domain proteins in anchoring the BAM complex to the peptidoglycan layer of diverse Gram negative bacteria.

We do not yet understand the subunit architecture of the BAM complex, but a clue to the docking mechanism for Pal comes from the interaction of Pal and TolB in *E. coli*. In *E. coli*, Pal binds to a β-strand from the propeller structure of the protein TolB, through an “induced fit”: a short segment in helix 3 of Pal is unwound and reforms as a β-structure paired with the TolB β-propeller [61]. The interaction of OmpA domain proteins like Pal and the BAM complex might be mediated via a similar induced fit mechanism through the BamB subunit, since BamB is predicted to be a β-propeller [24], [25], which would make BamB a crucial bridging element in the docking of BamA to peptidoglycan.

In order to receive substrate proteins in an “assembly competent” state, the mitochondrial SAM complex is positioned closely to the protein transporter, the TOM complex [62]. By minimizing the periplasmic distances involved, Pal might likewise help position the BAM complex in the proximity of the bacterial SecYEG protein transporter, to improve the efficiency of substrate protein transfer from the inner to outer membrane.

## Supporting Information

Figure S1 Immunoblot of Caulobacter cell lysate probed with anti-BamA and preimmune sera.
(0.03 MB PDF)
