# Supplementary material for: A Modular BAM Complex in the Outer Membrane of the α-Proteobacterium Caulobacter crescentus
Source: PLoS One. 2010 Jan 8;5(1):e8619. doi: 10.1371/journal.pone.0008619 (PMC2797634; doi:10.1371/journal.pone.0008619)
Supplement: Figure S1 — Immunoblot of Caulobacter cell lysate probed with anti-BamA and preimmune sera. (0.03 MB PDF) [file pone.0008619.s001.pdf]

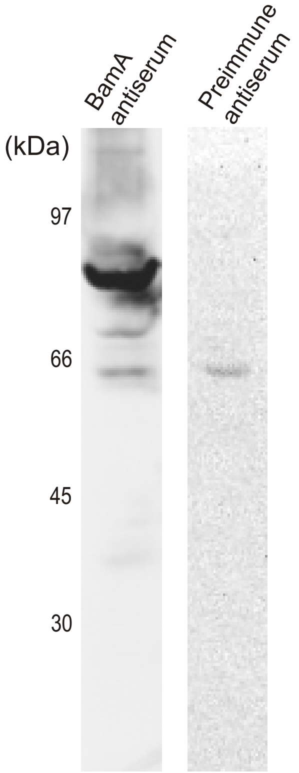

Supplementary Figure S1: Immunoblot of *Caulobacter* cell lysate probed with BamA antisera and pre-immunisation bleed.
